# Parents’ recalled experiences of the child centred health dialogue in children with overweight: a qualitative study

**DOI:** 10.1186/s12913-023-09308-8

**Published:** 2023-03-27

**Authors:** Malin Åsberg, Mariette Derwig, Charlotte Castor

**Affiliations:** 1 Vårdcentralen Staffanstorp, Södergatan 2, Staffanstorp, 245 31 Sweden; 2 Faculty of Medicine, Department of Health Sciences, Lund University, BOX 157, Lund, 222 40 Sweden

**Keywords:** Child centred, Childhood obesity prevention, Family-based, Primary care

## Abstract

**Background:**

Because overweight and obesity are still increasing and prevention of childhood obesity is more likely to be effective when initiated in preschool children, the Child Health Service in the south of Sweden developed a structured child-centred health dialogue model targeting all 4-year-old children and their families. The aim of this study was to describe parents’ recalled experiences of this health dialogue in children with overweight.

**Methods:**

A qualitative inductive approach with purposeful sampling was used. Thirteen individual interviews with parents (including 11 mothers and 3 fathers) were conducted and analysed with qualitative content analysis.

**Results:**

The analysis resulted in two categories: ‘A valuable visit with a subtle individual impact’ that described parents’ recalled experiences of the health dialogue and ‘There is a complex interaction between weight and lifestyle’ that reflected the parents’ perceptions of the relationship between their children’s weight and lifestyle.

**Conclusions:**

Parents recalled the child-centred health dialogue as important and described discussing a healthy lifestyle as one of the obligations of the Child Health Service. Parents wanted confirmation that their family lifestyle was healthy; however, they did not want to discuss the relationship between their family lifestyle and their children’s weight. Parents expressed that when their child followed the child’s growth curve, then this indicated healthy growth. This study supports using the child-centred health dialogue as a model to provide structure for discussing a healthy lifestyle and growth but highlights the difficulties of discussing body mass index and overweight, especially in the presence of children.

**Supplementary Information:**

The online version contains supplementary material available at 10.1186/s12913-023-09308-8.

## Background

Overweight and obesity are increasing among preschool aged children [1]. Obesity is associated with a range of physical and psychological health problems in children [2] and obesity in children often persists throughout adulthood [3]. Prevention of childhood obesity is more likely to be effective when interventions are initiated in preschool children or even earlier [4] and parents request interventions that can be applied in the preschool years, a period in which children’s behaviours and habits are shaped [5]. Both parents and professionals may have difficulty identifying preschool aged children with overweight and obesity [6]; therefore, body mass index (BMI) and the International Obesity Task Force (IOTF) standards a used to define ‘overweight’ and ‘obesity’ in growing children [7]. Swedish national data from 2018 based on 105,445 four-year-olds showed that the prevalence of overweight was 9% and the prevalence of obesity was 2% [8], which are comparable to the prevalence estimates of overweight and obesity among 2- to 7-year-olds (2006 to 2016) in other European countries (Netherlands, 10%; France, 11%; and Belgium, Finland and Hungary, 12%) [9].

In Sweden, the Child Health Service (CHS) reaches almost every child aged 0–5 years and their families. Nurses and physicians provide a package of healthcare visits to promote good health for all children during the early years, with extra visits according to need [10]. The 60-minute 4-year health visit includes measuring height and weight, calculating BMI, and testing vision, hearing, speech, motor skills and psychological development. The visit also involves a health dialogue and identifies overweight and obesity using the IOTF definitions [11]. However, nurses working within the CHS lack an evidence-based model to apply to children with overweight and obesity. Previous studies have shown that nurses described feelings of insecurity of the best approach to discussing overweight and obesity with children and their parents. They wanted appropriate guidance on how to raise the issue, and feared stigmatising children by linking them with childhood obesity [12, 13]. Discussing overweight with parents has been shown to be a delicate issue. Some parents find it difficult to believe that their child has overweight and dismiss the message [14], whereas other parents are concerned that talking to children about their weight might trigger low self-esteem or eating disorders [15]. Some studies have suggested that children with overweight, being labelled “as overweight” by their parents, may increase in BMI [16, 17]. Therefore, the Child Centred Health Dialogue (CCHD) has been developed as a structural approach that is designed to engage all family members in health promotion using sensitive non-judgmental communication skills and age-appropriate interactive, objective, and visual health promotion material. The CCHD consists of two parts: [1] a 10-minute universal part for all children and [2] a targeted part for families in which a child was identified with overweight. The universal part of the CCHD was implemented as part of the 4-year health visit and included a structured dialogue and eight illustrations that showed different aspects of a healthy lifestyle. A second tool, the BMI chart, was used to monitor each child’s growth and development and to identify overweight or obesity. At the end of the visit, each child received a storybook to read at home that had all the illustrations. The targeted part of the CCHD, the 45-minute ‘family guidance,’ was implemented 1–3 weeks after the universal 4-year health visit and was offered to children identified with overweight or obesity and their parents. This family guidance is inspired by the Standardized Obesity Family Therapy guidelines [18] and includes low-intensity treatment with a solution-focused approach that builds on family interactions as a basis for implementing and maintaining lifestyle changes.

A randomised controlled trial (RCT) that evaluated the CCHD’s effectiveness on weight gain in children with overweight produced a trend towards decreasing zBMI but was not statistically conclusive (*p* = 0.07) [19]. However, several core elements should be considered when performing intervention research in complex clinical settings, such as incorporating users’ experiences [20]. CHS nurses noted that involving children identified as having overweight was challenging, but that the CCHD training provided a structure that made it easier to guide children and their parents towards a healthier lifestyle [21]. Four-year-old children enjoyed participating in the health dialogue and were able to play an active role in the process, although their interpretations of the health messages sometimes differed from those of the adults [22]. Parents of children with normal weight reported that the universal part of the CCHD created supportive conditions for family members to actively participate, and they considered the health dialogue important and rewarding [23]. The aim of this study was to describe parents’ recalled experiences of the CCHD in children with overweight.

## Methods

### Study design

A qualitative inductive approach using semi-structured interviews with parents of children with overweight at the 4-year health visit. The Consolidated Criteria for Reporting Qualitative Studies (COREQ) were followed [24]. This study was part of an RCT that evaluated the effectiveness of the CCHD in promoting healthy weight, registered at ClinicalTrials.gov (2016721LUC3).

### Setting and participants

Thirty-five child health centres in southern Sweden were included and randomised and stratified according to the Care Need Index (CNI), which measures an individual’s need for care based on socioeconomic factors [25]. Nurses randomised to the intervention group were trained to use the CCHD approach, the relevant tools, the illustrations that increase child participation, and the BMI growth chart (21). Nurses were taught to discuss the child’s weight in a non-judgmental manner when overweight was identified, focusing on the child’s health and clarifying the importance of weight stabilisation [19]. A total of 238 children with overweight, according to the IOTF definition [7] who had been born between January 2013 and August 2014 were included in the intervention arm of the RCT [19].

The inclusion criterion for this study was parents of a child with overweight who participated in the intervention arm of the RCT. To achieve optimum variation within the sample, parents of enrolled children were selected strategically. First, one child from each centre allocated to the CCHD was randomly assessed: seven children from a centre in an area with high socioeconomic status and eight children from a centre in an area with low socioeconomic status, generating nine boys and six girls. Second, a letter was sent to the parents of these children explaining the purpose of the study and how the study would be performed. Thereafter, the first author (MÅ) contacted the parents, offered additional information and, for parents willing to participate, scheduled the date and location of the interview. Of the fifteen parents contacted by telephone, seven parents agreed to participate in the study, one declined, and seven did not answer repeated phone calls. To recruit more parents and ensure an even balance in CNI and child gender among the group, eight more children were selected. These children had participated in the intervention towards the end of the study period; therefore, their experience of the intervention was more recent. We followed the same procedure as before, and six parents responded and consented to participate.

### Data collection

Due to the pandemic, all interviews took place by video conference (Lund University Zoom, Lund University, Lund, Sweden) or by telephone, in accordance with the parents’ wishes. The interviews were carried out by the first author (MÅ) during November 2020 and April 2021. The interviews began by requesting background information; when an initial relation had been established, interviews continued using a semi-structured interview guide (Appendix 1). The interview guide includes opportunities for questions and suggestions for prompting more detailed responses when necessary. The first interview was conducted as a pilot interview and evaluated with the last author (CC). The interview guide fulfilled its purpose and remained unchanged; the pilot interview was included in the analysis. Interview durations were 10–35 min (median, 22 min). All interviews were recorded with the parents’ approval and transcribed verbatim.

### Analysis

Qualitative latent content was analysed using a structured stepwise method [26]. Two researchers (MÅ and CC) read the material several times to become familiar with the content. Meaning units were identified and thereafter condensed and coded based on their content. Similarities and differences among the codes were identified, and the codes were integrated into subcategories. In the latent analysis, the underlying meaning of the content was formulated into two categories (Table 1). All three authors were involved in the final evaluation of the results.

**Table 1:** Profile of the parents and children

	Parents	Children
	*N* = 14	* N* = 13
Female	11	5
Age (years)	33–46 (mean, 40.7)	6.7–7.9 (mean, 7.2)
University	10 *(2 missing)*	
Born in Sweden	12	
CNI * ≥ 0.93	7	
Timespan between 4-year health visit and interview (years)		2.5–3.9 (mean, 3.2)

* CNI, Care Need Index ≥ 0.93 indicates living in an area with low socioeconomic status

## Results

Parents of 13 children were included in the study (Table 1). Ten interviews were performed with the mother, two with the father and one with both parents. During two interviews, the other parent was present, prompting at times. Participants had different ages and educational levels. They were born in Sweden, Europe or outside Europe. Some parents lived in areas with high socioeconomic status (CNI < 0.93), whereas others lived in areas with low socioeconomic status (Table 2).

**Table 2:** Overview of meaning units, condensed meaning units, codes, subcategories and categories

**Meaning units**	**Condensed meaning units**	**Codes**	**Subcategories**	**Categories**
It might be good if the child is there, if you do it in the right way and have this with weight, the focus on weight I mean, that you do that in the right way so it does not become something dramatic, shameful, I do not think that is good.	Child participation can be good but important not to focus on weight as this could be dramatic and shameful	Child participation without focus on weight	Optimal child participation is complicated	A valuable visit with a subtle individual impact
Considering what I have read over the last few years; yes, it is literature describing the weights of adults, BMI is not a very good valuation… it is not widely representative of weights because circumstances can be so different.	I have read that BMI is not a good valuation.	Mistrust BMI	BMI is neither adequate nor relevant	A complex interaction between weight and lifestyle

Seven subcategories in two categories were identified. One category, ‘A valuable visit with a subtle individual impact,’ described parents’ recalled experiences of the health dialogue and the other category, ‘A complex interaction between weight and lifestyle,’ reflected the parents’ perceptions of the relationship between their child’s weight and lifestyle (Table 3).

**Table 3:** Subcategories and categories

Subcategories	Categories
One of many valuable health visits Illustrations as tools to involve the child Optimal child participation is complicated High expectations of the nurse	A valuable visit with a subtle individual impact
Following one’s growth curve is desirable BMI is neither adequate nor relevant A healthy lifestyle is natural but challenging to maintain	A complex interaction between weight and lifestyle

### A valuable visit with a subtle individual impact

Parents reported that visiting the CHS during their child’s early years was a valuable experience. Discussing a healthy lifestyle was perceived as a logical part of both the CHS visit during the child’s early years and the 4-year health visit that included the CCHD. The parents recalled that the illustrations were simple and easy for most children to understand. Knowing the nurse prior to the dialogue helped parents and children to take part in the conversation and to make changes to improve the family’s health. Changes that were made could initially go unnoticed but were discovered after observing changes in the child’s health.

#### One of many valuable health visits

Parents considered visiting the CHS as valuable and regarded the visits as checks to ensure the child was doing well. Parents expressed their overall gratitude and satisfaction with the CHS, and they were happy that the importance of a healthy lifestyle was highlighted. Sometimes, the 4-year health visit was recalled as a visit that focused on lifestyle and weight. At other times, it was difficult to distinguish the 4-year health visit from the other CHS visits because a healthy lifestyle had been discussed at several visits.

Parents reported that visits to the CHS seldom led to marked changes in their own family’s everyday life. They regarded change as unnecessary because they already had a healthy lifestyle and did not feel that the visits altered their behaviour. At the same time, parents recalled that the issues discussed during the visits were useful reminders for discussing lifestyle at home both between the parents and with the child. The parent could ask or remind the child what the nurse had said. Sometimes, this led to the parents eventually noticing changes. For example, the child stopped being constipated, even though they did not recall a specific alteration in lifestyle.

*We have different ways of doing things and different abilities but you want to do your best; so, if you’re given good advice, you try to follow it, and if the same advice comes from other sources, you are more likely to eventually make a change.* (Mother in interview no. 3, 7-year-old boy)

#### Illustrations as tools to involve the child

Parents recalled that the illustrations both facilitated and complicated the dialogue about a healthy lifestyle. When listening to their child discussing the illustrations with the nurse, the parents were impressed that their child knew so much about a healthy lifestyle and was able to reflect on it. At times, parents thought that the illustrations could also impede the conversation as the child struggled to understand the message that was illustrated or had trouble interpreting the broader meaning of an illustration. Parents hypothesised that making the illustrations more fun and richer in content might help to involve the child even more, although they knew that the simple layout was designed to be easily understood by all children.

*[The illustrations] were quite simple, weren’t they? But maybe that’s because they should participate, the 4-year-olds, so it’s important that [the illustrations] are easy to understand.* (Mother in interview no. 8, 7-year-old boy)

#### Optimal child participation is complicated

Parents expressed mixed feelings about involving the child in the conversation on a healthy lifestyle, especially if weight was discussed. They felt that the child listened more closely to the nurse than to the parent, even if both said the same thing. Furthermore, parents recalled that the child sometimes told the truth about less flattering aspects of family life, such as eating habits, which the parents were reluctant to talk about. At times, the parents recalled that involving the child in the discussions created difficulties. For example, the child may not focus on the issue that the adults were concerned about, or they may become uncomfortable if they felt that too much focus was on them. However, parents reported that after the visit their child was pleased that they were able to participate and was motivated by the fact that someone had listened to what they had said.

Parents worried that focusing on weight might generate feelings of guilt or even increase the risk of the child developing eating disorders. These worries could make it difficult for parents to talk freely in the presence of their child if the child was considered mature enough to understand what was said but not mature enough to understand the meaning of the conversation.

*Many kids simply accept their situation, so if someone actually asks them questions and allows them to express themselves, they might find this empowering: ‘if I say something, someone listens and it has an influence’ and then they will continue with that; so, there the illustrations were very good… however, these questions at the end about BMI, there you might not talk to the child. At that age they shouldn’t think about themselves in that way…in the end it’s judging their body*. (Mother in interview no. 3, 7-year-old boy)

#### High expectations of the nurse

Dialogue with parents regarding a healthy lifestyle was considered a basic and important part of the CHS’s role and a good method of social education. Parents expected the nurse to provide comprehensive and specific information on healthy living, with a particular focus on each family’s lifestyle. This was facilitated when parents became familiar with their CHS nurse, and changing a family’s CHS nurse was considered counterproductive.

Parents recalled being grateful for information that had been prepared by the nurse so that they could focus on assimilating the advice. Parents recalled that they were attentive to the CHS nurse and expected the advice provided to be accurate and up to date. Parents wanted the nurse to confirm that their own lifestyle was healthy and if the nurse did not suggest any changes, parents assumed that everything was in order.

Parents emphasised the importance of the nurse being able to assess each family’s lifestyle within the appropriate cultural context and provide useful information to those who needed it. Parents preferred to receive information via dialogue with the nurse or as specific instructions. The parents also asked for written information because they did not remember everything that was said during the visit and some parents experienced language barrier difficulties with the spoken word.

*Given our interest, I wanted the information provided to be comprehensive and up to date. I did not want to settle for what we have known for 40 years about children’s diets.* (Mother in interview no. 10, 7-year-old girl)

### A complex interaction between weight and lifestyle

Parents recalled being suspicious and mistrustful of discussions about weight because they did not think their children had overweight. The relevance of measuring BMI in young children was questioned, as was the usefulness of labelling young children as having overweight. The parents preferred the regular growth chart to see whether their child followed the appropriate growth curve. They wanted confirmation that they already had a healthy lifestyle but did not want to talk about lifestyle in relation to weight.

#### Following one’s growth curve is desirable

When parents referred to the growth chart, they stated that when their child followed the appropriate growth curve everything was okay. Parents thought that at some ages children were allowed to be over the growth curve on the growth chart. Often the child was referred to as large at birth, a little above their growth curve, or not having overweight anymore. At times, parents used expressions such as chubby, robust or sturdy, or said that the child did not look overweight or that the child was overweight but that this was only noticeable when they were compared with other children.

*He has always been a chubby child you might say, but he has always followed his growth curve; he has always been shorter and weighed a bit more [than some other children], but not to a worrying extent.* (Mother in interview no. 11, 7-year-old boy)

Parents did not recall nurses using the word overweight at the 4-year health visit but instead recalled that the nurse had said that there was nothing to be concerned about and that the child was of normal weight. They described that this made them feel that there was nothing to worry about.

#### BMI is neither adequate nor relevant

Despite their wish to focus on a healthy lifestyle rather than weight, parents said they recalled wanting to know whether their child had overweight and suggested the height and weight curve as a basis for these conversations. At the same time, parents expressed a general mistrust of the concept of BMI and recalled being surprised and upset when the CHS measured a child’s BMI, either during their own visit or when this was reported by other parents. Using BMI was experienced as problematic. Parents understood that BMI was used to determine whether an individual had overweight but believed that individuals had different preconditions and muscle masses, and that these factors should be taken into account. To illustrate, parents described themselves as having a high BMI without having overweight because they had an appropriate balance between muscle and fat. Therefore, parents stated that while their child was growing, there was no need to measure BMI.

*To put a BMI growth chart on a child is a little bit too much, a child develops all the time, they shoot up in height, stop growing, and then all of a sudden your child is overweight, obese maybe and then you need to put your child on a diet? No, you should not put a child on a diet! The child should be allowed to follow the growth chart.* (Mother in interview no. 2, 7-year-old boy)

#### A healthy lifestyle is natural but challenging to maintain

Parents considered a healthy lifestyle to be about finding a balance in their everyday family life. They recalled trying to focus on aspects that promote good health, such as what to eat and what sports the children could participate in rather than discussing weight in front of their children. Various factors affected parents’ perceptions of a healthy lifestyle and the best ways to achieve this. Bad memories and body shaming experiences from a parent’s childhood could have negative effects, as could their own experience of having overweight, having an eating disorder, or having a preoccupation with weight or dieting. Parents recalled often trying to conceal these thoughts because they did not want their children to develop similar problems or perhaps provoke eating disorders.

*At home, we are really cautious, we do not want the child to lose confidence (...) We try to never compare them at home. I am overweight, I must rethink, I must change my eating habits, I must start exercising; so the kids come along, they want to be like their parents, but you never say these things to the child.* (Father in interview no. 7, 7-year-old girl)

One difficulty in maintaining a healthy lifestyle was that parents were not always able to control their child’s environment. For example, grandparents may not adhere to family rules and may give the child cookies without the parents’ permission.

Parents thought they had healthy lifestyles, and they wondered what they were doing wrong and what they could have done differently if their child was identified as having overweight. They found it difficult to understand why their child’s BMI was greater than normal when the nurse had confirmed that they were doing everything right. They compared their child with his/her siblings who had normal weight and shared the same lifestyle. This was used to justify continuing with the current lifestyle.

*She and her sister have gained a lot of weight and we’re working on it, but you can’t make drastic changes with kids and you don’t want to put them on a diet. I feel we can hardly have candy on Saturdays or take a cake in the middle of the week and I think you should be able to do that… I grew up with a bad attitude towards my body, but I have managed to deal with it.* (Mother in interview no. 4, 8-year-old girl)

## Discussion

This study describes parents’ recalled experiences of the CCHD for children with overweight. One significant finding was that parents of children with overweight did not have specific recollections regarding the CCHD. Specific memories of each visit had faded and none of the parents recalled feeling upset or being questioned about their lifestyle during their visits; however, the parents retained positive impressions of the visits.

A possible explanation for this observation is that the nurses trained in CCHD might have tailored discussions regarding each child’s weight and each family’s lifestyle to suit each family. The use of positively framed messages, such as the CCHD illustrations, is considered a neutral method for discussing sensitive topics such as lifestyle and can tailor dialogue to meet a family’s needs [21, 27]. The CCHD trained nurses noted that deciding how and when to raise the issue of overweight was difficult. Nurses felt responsible for helping the child and family to achieve a healthier lifestyle, but on the other hand, they wanted to be sensitive to the perceived needs of the child and the family [21]. Parents appreciated the importance of talking about a healthy lifestyle during their visits to the CHS. They thought that discussing lifestyle was one of the obligations of the CHS. However, the parents stated that they already had healthy lifestyles. Therefore, they believed that the health messages from the CHS were most applicable to *other* families, as described in an English focus group study among parents of 4- and 5-year-old children who received feedback on their child’s weight [14].

Overall, parents were positive about including their child in the CCHD and were impressed with their child’s ability to participate in the discussion and reflect upon the health messages. In other words, the illustrations improved the children’s health literacy, allowing them to understand and reflect upon the messages and make healthy choices for themselves. This observation is consistent with a recent systematised review of research literature in which storytelling, visual materials and reflection were described as core elements in supporting health literacy development and providing motivation to make healthy choices [28]. Sometimes, parents expressed that their children misinterpreted the health messages associated with the illustrations and believed that the illustrations hindered their children’s participation in the health conversation. That adults and children interpreted the illustrated health messages in different ways has been confirmed by a study elucidating children’s experiences of the CCHD [22]. The study was based on observations and interviews with children who had recently taken part in the CCHD; it showed that children’s and adult’s interpretations of the illustrations sometimes differed and that children may become preoccupied with their own interpretations and do not focus on the messages that the nurses wanted to discuss.

Similar to a Norwegian study involving parents of children aged 2.5–5.5 years who had been identified as having overweight [29], we found that parents were reluctant to include their children in the health dialogue if the word overweight was being used, due to the risk of eliciting feelings of guilt or stigmatising the children. This is corroborated by another Swedish study involving parents of 3- to 7-year-old children with overweight or obesity [15], which stated that some parents did not wish to discuss the topic of overweight in the presence of their children to protect their children from weight conversations with negative connotations. However, previous research has shown that children ask for age-appropriate information on their own health status and according to the United Nations Convention on the Rights of the Child, children are entitled to such information. A study in which children with overweight who were aged 8–13 years participated in a health course suggested that participation in a non-judgmental health dialogue could help children to improve their health. These children articulated that the course helped them to develop strategies to cope with challenges and improve health behaviours in their everyday lives [30]. Further studies will be needed to determine whether very young children with overweight should be involved in such discussions. This is particularly important because children are end users of interventions to prevent childhood obesity and should be involved in the early stages of intervention development as much as possible [22].

Moreover, parents thought that measuring a child’s BMI was unhelpful, and they wanted to use a regular growth chart instead. There was a mistrust of measuring BMI in general and especially of using BMI to identify children with overweight. However, parents did want to know whether their child had overweight. Some parents considered BMI a complex measurement, rather than a means of highlighting the relationship between a child’s height and weight. Other studies have indicated that parents have difficulty understanding BMI charts [31, 32]. Parents in this study communicated that children who followed their growth curves on the growth chart were exhibiting healthy growth and could not have overweight, regardless of the relationship between height and weight. This may be an important insight for CHS nurses who could implement the parents’ confidence in their children’s growth curves by illustrating each child’s growth not only using the weight and height growth charts but also the BMI growth chart from an early age, when parents visit the CHS.

Over the last few decades, there has been a prevailing assumption that making parents aware that their child has overweight is a necessary step in initiating appropriate changes in lifestyle [33, 34]. However, some studies have found that parents’ awareness of their children having overweight does not create positive changes and that the children’s BMIs may actually increase [16, 17]. The parents in this study did not consider their children overweight and did not remember the nurses telling them that their children had overweight. One explanation might be that the nurses did not use the word overweight but discussed the child’s weight development in a positive manner, engaging the child and the parents in a non-judgmental dialogue about health and health behaviours, in accordance with the CCHD guidelines. There is evidence that healthy behaviours do not automatically develop when unhealthy behaviours are reduced but instead require positive reinforcement [35, 36]. For example, public health media messages may be motivating when they are formulated in a positive manner and focus on making healthy behavioural changes [37]. Another explanation is that nurses did not raise the issue of overweight in the presence of the child because they, like the parents, feared exacerbating the stigma of childhood obesity [12, 13]. These observations emphasise the need to train health professionals how to communicate weight-management messages and the need for initiatives that help to end weight stigma and discrimination on the societal level [38].

This study had both strengths and limitations. There was variation in the ages, gender, educational levels and CNIs of parents recruited, which enhanced the richness of our data. However, including background data on the parents’ weights and lifestyle factors could have improved the credibility of our findings further. All parents were contacted several times and all of those who responded wished to participate in the study, except one parent who did not remember their health visit.

Because several years (2–4 years) had passed between each child’s 4-year health visit and our study, our results highlight the long-term effects of the CCHD on parents.

All authors have extensive clinical experience of conversations with parents and children in various healthcare situations and are well acquainted with the CHS. The first (MÅ) and last (CC) authors were not involved in the RCT or in conversations on lifestyle or weight with families within the CHS. To increase confirmability, our analysis was performed with frequent reference to the study aims and the interview guide, and the method described by Graneheim and Lundman was followed [26]. The second author, who was the principal investigator in the RCT, was not involved in the primary analysis of the interviews, to minimise the risk of bias. The authors discussed the analysis until they agreed completely on the final categories. Quotations from parents’ statements are included in the results to help the reader to assess the study [39].

## Conclusions

Parents recalled the CCHD as important and described discussing a healthy lifestyle as one of the obligations of the CHS even if they believed this was not always relevant for their own family. Parents considered their children’s participation in the CCHD as valuable and were impressed at their children’s ability to reflect on their own health. In this way, the CCHD aligns with the aims of the CHS in promoting child health and with the United Nations Convention on the Rights of the Child, which states that children have the right to actively participate in their own healthcare. This study is consistent with previous studies that found parents wanted confirmation that their own family lifestyle was healthy, but preferred not to discuss the relationship between their own family lifestyle and their children’s weight. BMI and overweight were considered potentially stigmatising concepts that were not conducive to promoting a healthy lifestyle and should not be discussed in the presence of children. Parents believed that if their child followed the child’s growth curve on the growth chart, then this indicated healthy growth. This insight may inform weight related conversations. This study indicates that the CCHD can be used to provide a structure for discussing a healthy lifestyle and growth, but more research is needed to understand the best methods for broaching the topic of overweight with parents in the presence of the child recognising the rights of the child.

## Electronic supplementary material

Below is the link to the electronic supplementary material.

Supplementary Material 1

## Data Availability

The data generated and analysed in this study will not be shared, to maintain participants’ confidentiality, but they are available from the corresponding author in response to a reasonable request.

## List of abbreviations

BMI: body mass index
CCHD: Child Centred Health Dialogue
CHS: Child Health Service
CNI: Care Need Index
RCT: randomised controlled trial.
