# Supplementary material for: Parents’ recalled experiences of the child centred health dialogue in children with overweight: a qualitative study
Source: BMC Health Serv Res. 2023 Mar 27;23:289. doi: 10.1186/s12913-023-09308-8 (PMC10045090; doi:10.1186/s12913-023-09308-8)
Supplement: Supplementary file 1 — Supplementary Material 1 [file 12913_2023_9308_MOESM1_ESM.docx]

**Introduction**Acknowledge participation, repeat study purpose and stress that participation is voluntary and can be interrupted without stating a reason. Make time for questions and for obtaining consent.

**Introductory question**Do you remember your nurse talking about lifestyle habits and their importance at your child´s 4-year visit to the Child Health Service and what can you tell me about this visit? Who participated in the conversation(s)?

Topics to include during the interview, by direct questioning or spontaneously.

Parents’ expectations and experiences of the health conversation: universal and family-guided parts.

If and how weight and overweight were mentioned in the conversation and parent´s experiences/reactions to this. Parents’ experiences/recollections of the child´s participation during the health conversation.

How illustrations and body mass index charts were perceived and how were they used

How the conversation affected the family´s diet and physical activity habits over time.

**Actions to prompt further response**Questions: Can you elaborate? Could I ask you to describe this further? Can you tell me more?

A copy of the storybook that was given to the child at the time of the CCHD. (This was referred to when needed and shown if necessary, during the interviews performed trough video calls)

**Finishing question**Is there anything that we have not touched upon that you would like to add?

**After the interview**Offer of follow-up calls, including contact details for support.
